# Sudden unilateral hearing loss and vertigo following isolated cerebellar hypoperfusion without infarction due to vertebral artery dissection

**DOI:** 10.1186/s12883-022-03024-2

**Published:** 2022-12-19

**Authors:** Young Seo Kim, Ali S. Saber Tehrani, Hak Seung Lee

**Affiliations:** 1 Department of Neurology, Institute of Wonkwang Medical Science, Wonkwnag University School of Medicine, Iksan, South Korea; 2 Department of Neurology, Jeonbuk Regional Cardiocerebrovascular Center, Wonkwang University Hospital, 895 Muwang-ro, Iksan, Jeollabuk-do 54538 South Korea; 3 Department of Neurology, Division of Neuro-Visual and Vestibular Disorders, Johns Hopkins University School of Medicine, Baltimore, MD USA

**Keywords:** Acute hearing loss, Vertigo, Hypoperfusion, Infarction, Perfusion-weighted imaging, Case report

## Abstract

**Background:**

The definition of sudden sensorineural hearing loss (SSNHL) is broadly accepted as acute sensorineural hearing loss of more than 30 dB over at least three consecutive frequencies in a pure-tone audiogram (PTA). Acute audiovestibular loss is common with ischaemic stroke in the territory of the anterior inferior cerebellar artery (AICA). However, cases in which SSNHL and vertigo occur with hypoperfusion alone are very rare. We describe a patient who developed unilateral SSNHL and vertigo as initial symptoms caused by cerebellar hypoperfusion by vertebral artery (VA) dissection without the occurrence of infarction.

**Case presentation:**

A 51-year-old man suddenly developed acute hearing loss (AHL) in his left ear and vertigo. On neurological examination, he had vibration-induced right-beating nystagmus and left-beating nystagmus after a head-shaking test. Additionally, he had apogeotropic nystagmus during head turns to either side. The head impulse test (HIT) was normal. PTA showed mild unilateral SSNHL in the left ear. Diffusion-weighted imaging (DWI) and perfusion-weighted imaging (PWI) showed territorial perfusion deficits in the left posterior inferior cerebellar artery (PICA) and anterior inferior cerebellar artery (AICA) without infarction. Two months later, the patient had no vertigo but still had hearing impairment in his left ear. A follow-up PTA documented persistent unilateral SSNHL in the left ear. Additionally, perfusion computed tomography (CT) showed that perfusion deficits remained in the left cerebellum along the PICA and AICA territories.

**Conclusions:**

Our case highlights a case of AHL and vertigo presented by isolated cerebellar hypoperfusion without infarction. It is necessary to consider the possibility of a central cause in patients with AHL and vertigo, and it is important to confirm this possibility through brain magnetic resonance imaging (MRI), including PWI, and magnetic resonance angiography (MRA).

## Background

Sudden sensorineural hearing loss (SSNHL) is a sudden and unexplained hearing loss that is frequently unilateral. The US National Institute for Deafness and Communication Disorders defines SSNHL as an idiopathic hearing loss of at least 30 dB over at least 3 test frequencies within 72 hours [1]. The aetiology and pathogenesis of SSNHL are unclear but they may be multifactorial. Several factors such as inflammation, infection, trauma, aging (presbycusis), ototoxic drugs, autoimmune conditions, rupture of the labyrinthine membrane, and vascular insufficiency may be involved [2].

The evidence suggests that the development of SSNHL can be an initial warning sign of vascular events, such as thromboembolism, vasospasm, and insufficient blood circulation [2, 3]. Audiovestibular manifestations, such as acute hearing loss (AHL) and vertigo are usually caused by occlusive diseases of the vertebrobasilar system. Among the vascular events, territorial infarction of the anterior inferior cerebellar artery (AICA) caused by thrombosis or embolism is the leading cause. In a previous report, only 1% of patients with AHL experienced a non-AICA territory vertebrobasilar ischaemic stroke [4].

There have been reports of SSNHL related to infarction, especially AICA territorial infarction, and although infarction was subsequently observed, it occurred at a site different from the hypoperfusion area; furthermore, it is known that cases in which SSNHL and vertigo occur with hypoperfusion alone are very rare. We describe a patient who developed unilateral SSNHL and vertigo as initial symptoms caused by cerebellar hypoperfusion by vertebral artery (VA) dissection without the occurrence of infarction.

## Case presentation

A 51-year-old man presented with a sudden loss of hearing in his left ear. He also noted vertigo and imbalance. Approximately three times over the previous week, the patient had experienced transient hearing loss and vertigo lasting between 5 and 10 minutes. The patient had a history of hypertension and heavy smoking but he did not have a history of hearing impairment, head trauma, central nervous system infection, autoimmune diseases, or exposure to ototoxic drugs.

**Fig. 1:**
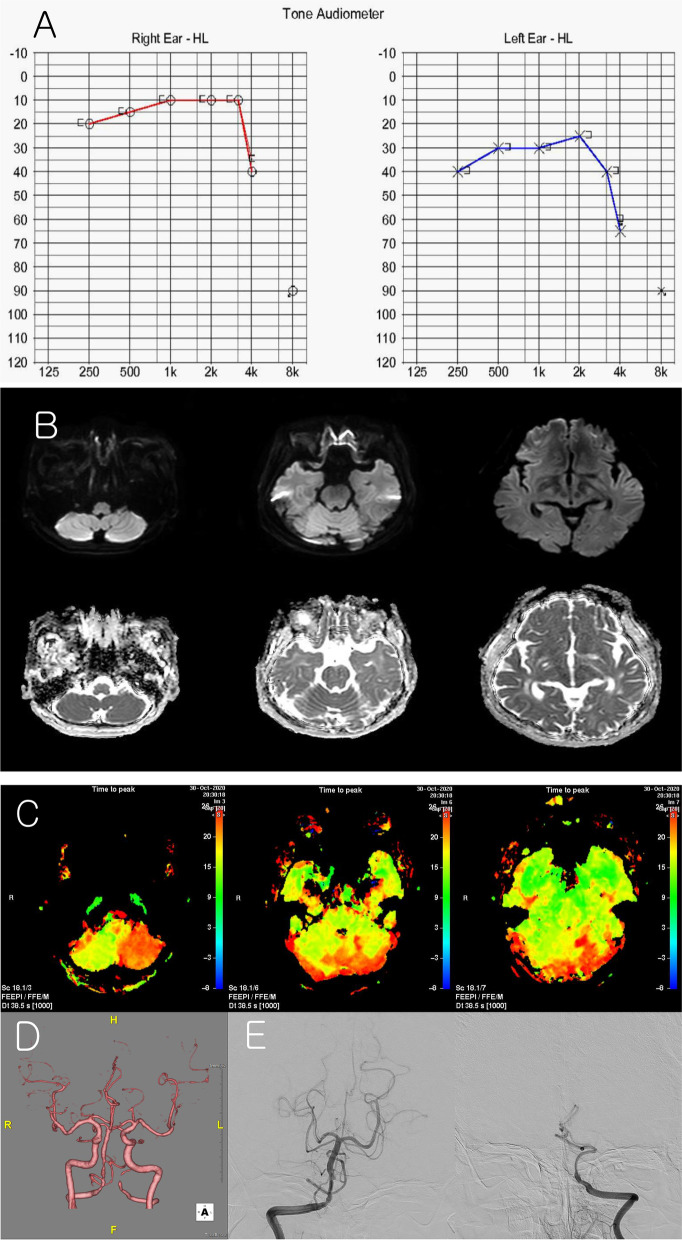
Pure-tone audiogram (**a**) showed a mild unilateral sensorineural, pure-tone average of 37 dB (dB) in the left ear. Diffusion-weighted imaging and apparent diffusion coefficient map (**b**) showed normal findings. Perfusion-weighted imaging (**c**) revealed left PICA and AICA territorial perfusion deficits without infarction. Brain CT angiography (**d**) revealed occlusion of the left post-PICA intracranial VA and multifocal stenosis of the right intracranial VA, indicating that both VA dissections were confirmed by conventional angiography (**e**)

On neurological examination, he had vibration-induced right-beating nystagmus and left-beating nystagmus after a head-shaking test. Additionally, he had left-beating nystagmus in the right roll test and right-beating nystagmus in the left roll test. The head impulse test was normal. Analysis of blood contents, including complete blood count, serum electrolytes, liver and kidney function indicators, and high-sensitivity C-reactive protein (hsCRP), were normal, except for an increased LDL-cholesterol level (130 mg/dL). Pure-tone audiogram (PTA) showed a mild unilateral sensorineural, pure-tone average of 37 dB (dB) in the left ear (Fig. 1A).

Magnetic resonance imaging (MRI) of the brain was performed 9 hours after the last event. Diffusion-weighted imaging (DWI) and apparent diffusion coefficient (ADC) maps showed normal findings (Fig. 1B). Perfusion-weighted imaging (PWI) (Fig. 1C) showed territorial perfusion deficits in the left posterior inferior cerebellar artery (PICA) and anterior inferior cerebellar artery (AICA) without infarction. Brain CT angiography (CTA) (Fig. 1D) revealed occlusion of the left post-PICA intracranial VA and multifocal stenosis of the right intracranial VA, indicating that both VA dissections were confirmed by conventional angiography (Fig. 1E).

**Fig. 2:**
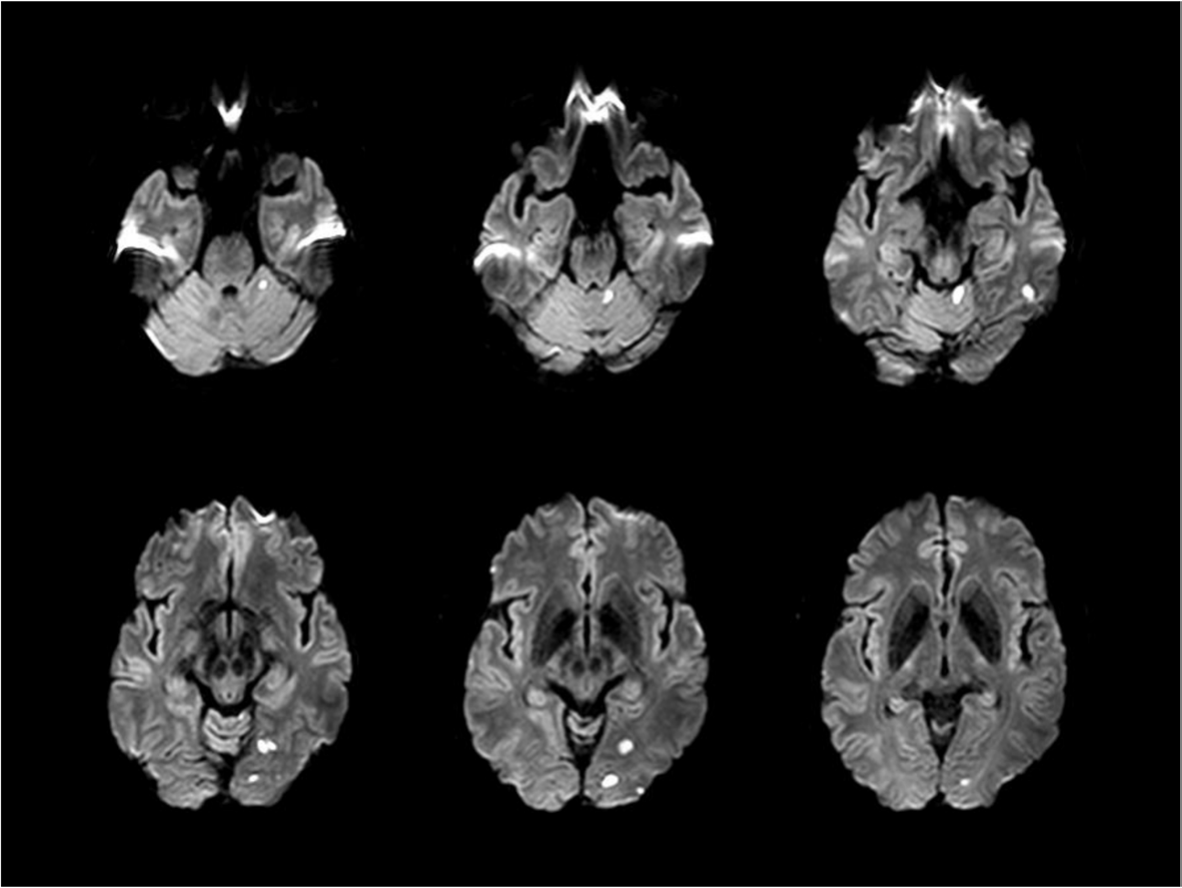
Follow-up diffusion-weighted imaging and apparent diffusion coefficient mapping performed 3 days after onset demonstrated multifocal infarctions along the posterior circulation involving the upper cerebellum and occipital lobe and not the PICA and AICA territories

**Fig. 3:**
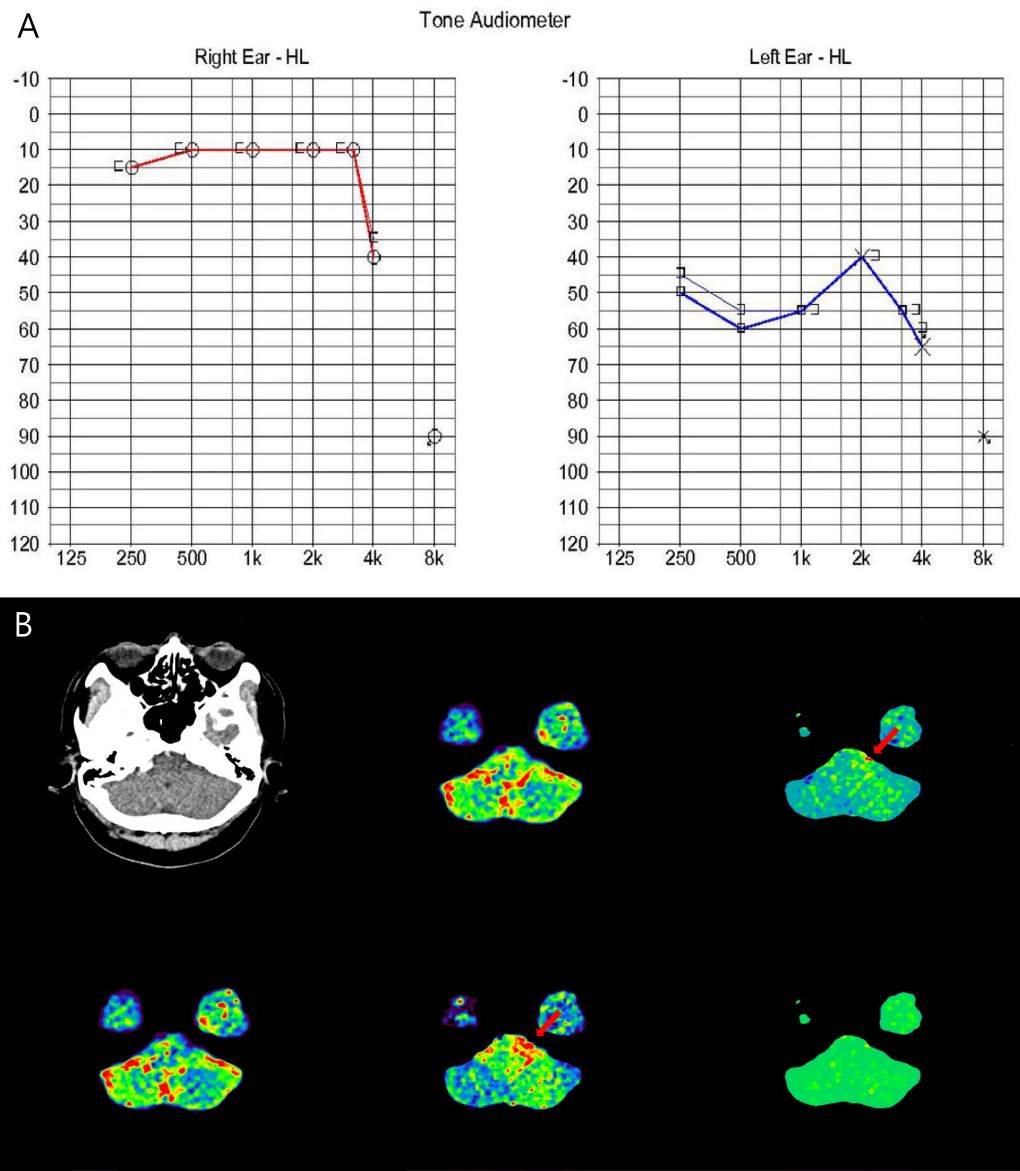
Pure-tone audiogram 2 months later (**a**) documented persistent unilateral sensorineural, pure-tone average of 53 dB in the left ear. Additionally, perfusion CT (**b**) showed that the perfusion deficits remained in the left cerebellum along the PICA and AICA territories (red arrow)

We started treating the patient with 75 mg of clopidogrel and 40 mg of atorvastatin. After starting treatment, his vertigo gradually improved, but his SSNHL did not change. Follow-up DWI and ADC mapping performed 3 days after onset showed multifocal infarctions along the posterior circulation involving the upper cerebellum and occipital lobe, not the PICA and AICA territories (Fig. 2). Two months later, the patient had no vertigo but still had hearing impairment in his left ear. A follow-up PTA documented a persistent unilateral sensorineural, pure-tone average of 53 dB in the left ear (Fig. 3A). Additionally, perfusion computed tomography (CT) showed that perfusion deficits remained in the left cerebellum along the PICA and AICA territories (Fig. 3B).

## Discussion and conclusions

Hearing impairment seriously affects not only the physical and mental health of patients but also the social life and activities of daily living. Circulation system disorders, such as ischemic stroke and myocardial infarction have closely related to SSNHL [2]. Because the blood supply to the audiovestibular system arises from the vertebrobasilar circulation, hearing loss and/or vertigo are also common complications of vertebrobasilar ischaemic stroke (VBIS). The most common territory of infarction related to acute SSNHL on brain MRI is in the distribution of the AICA because the internal auditory artery, which mostly originates from the AICA, is the main artery for vascular supply to the inner ear with minimal collaterals [5]. Several reports on the frequency of acute hearing loss associated with VBIS. In a retrospective analysis of patients with VBIS, approximately 1.4% (7/503) of patients had bilateral hearing loss, documented by audiometric examination [6]. In addition, several reports have found that sudden deafness with audiometric data can be a prodrome of vertebrobasilar insufficiency (VBI), and they mentioned that it was an important clue for the detection of AICA infarction [7, 8].

In this case, the patient had intermittent unilateral hearing loss and vertigo 1 week before the onset of symptoms. This shows that our patient was in the same situation described with infarction of one of the eight subgroups of the AICA area: sudden onset of audiovestibular syndrome with vertigo and hearing loss that is occasionally preceded by a prodromal episode(s) of audiovestibular disturbance [5, 9]. Our patient had a history of hypertension and heavy smoking, and the cause of AHL likely had a vascular origin. This is consistent with a previous report that a vascular cause should be highly suspected in patients with combined audiovestibular loss and prolonged vertigo [5].

In this case, overall hypoperfusion, including in the AICA and PICA territories observed with PWI, is thought to be the cause of the AHL and vertigo due to the prodromal symptoms of infarction. However, the pattern of cerebral infarction observed in follow-up DWI was not the previously known AICA, and the PICA infarction showed the possibility that isolated cerebellar hypoperfusion alone can induce persistent AHL. There are few studies on AHL and isolated cerebellar hypoperfusion. In one study involving acute transient vestibular syndrome (ATVS), The ipsilesional VA stenosis or occlusion may cause unilateral cerebellar hypoperfusion in patients with ATVS. Almost 80% of patients with cerebellar hypoperfusion revealed luminal abnormality or corresponding VA hypoplasia [10]. In addition, a recent study showed that ATVS patients associated with VA stenosis or hypoplasia had a higher risk for posterior circulation ischaemia [10, 11]. VA hypoplasia is thought to be more vulnerable to vertebrobasilar ischaemia, especially when conventional vascular risk factors coincide [11]. In another study, a decrease in AICA blood flow in a patient with AHL was observed on 3-dimensional stereotactic surface projection single photon emission computed tomography [12]. As in this case, these studies show that isolated cerebellar hypoperfusion can cause AHL even if cerebellar infarction does not occur.

There are some reports that an initial normal MRI does not rule out vascular aetiology [13]. However, as in this case, the locations of the infarcted area were different from the hypoperfusion areas, and the locations were not related to AHL. These results imply that the multifocal infarcted area has been affected by embolism since VA dissection, and as a result, the initial PWI and later DWI show different lesions, which are also associated with the progression of VA dissection. Therefore, we think that this is the first case to show that AHL can occur with continuous hypoperfusion even if infarction does not occur.

In a study of patients with AHL due to posterior circulation ischaemia, nearly 80% of the patients who were followed for at least 1 year after the onset of sudden deafness due to vascular cause (mainly cerebellar infarction of the AICA territory) had a partial or complete recovery of hearing. Multivariate analysis revealed that multiple vascular risk factors for ischaemic stroke and profound hearing loss had poorer outcomes regarding recovery from AHL [8]. Although there have been no systemic reports on the factors indicating poor outcomes in patients with AHL due to vascular causes, the improvement rate of hearing loss in patients with severe hearing loss had a remarkably poorer outcome than the patients with less than severe hearing loss (40% vs. 94%) [8]. In this case, the hearing loss persisted without recovery even after 2 months, and follow-up perfusion CT showed that hypoperfusion was still present in the left AICA territory.

Although the exact mechanism of the long-term outcome of AHL associated with VBIS is not clearly understood, the maximum improvement is usually seen within the first 2 weeks after the onset of hearing loss [14]. The prognosis for recovery is best when the patients are detected early, recovery begins within 2 weeks, and there is a mild loss with an upwards-sloping audiogram without vertigo. The previous study has reported the prognosis for recovery mainly depends on a mild loss at presentation with an upwards-sloping audiogram and an absence of vertigo. Patients who have flat or downsloping audiograms with an AHL of less than 90 dB are likely to have variable outcomes. Old age and the presence of vertigo can affect the poor prognosis [15]. In this case, the patient visited the hospital relatively late after the first symptoms occurred, and the fact that he had flat or downsloping audiograms with AHL of less than 90 dB and it was accompanied by vertigo is presumed to be related to his continued hearing loss. We speculate that his persistent hypoperfusion is also related to his continuation of hearing loss. Therefore, it can be hypothesized that improvements in AHL do not naturally occur over time and only occur when hypoperfusion is improved.

The HINTS examination (head impulse test, direction-changing nystagmus, test of skew) provides the earliest collection of physical examination findings developed to reliably rule out a central cause of hearing loss from a peripheral cause [16]. Any other findings should raise concern for a central cause, and a stroke workup should be performed. For central causes, HINTS is 96.8% sensitive and 98.5% specific compared with 14.3% falsely negative MRI in the first 48 hours [17]. HINTS Plus is the same as HINTS but with the addition of audiometry [18]. New-onset hearing loss with acute vestibular syndrome was identified as an additional predictor of stroke based on a 2013 population-based study: its addition increased the sensitivity for stroke detection to 99% [17].

Although HINTS has been used academically for more than 10 years, There are still limitations to practical use in clinical practice. Many physicians in acute emergency settings tend to rely on neuroimaging because they lack the confidence to use their examination and interpretation of eye movements to make a critical decision about whether a patient has had a stroke. This means that the usefulness of HINTS strongly depends on the level of expertise of the person performing it [19]. Additionally, a formal audiogram is not feasible in most emergency department settings, and physicians should rely on bedside testing. If a hearing assessment cannot be obtained in the emergency setting, an outpatient audiogram should be arranged to aid in risk stratification and can confirm the patient’s hearing status only after the stroke is diagnosed. Although normal HIT and AHL were observed in this case, it could not be regarded as a finding completely suitable for HINTS and HINTS Plus.

We treated the patient with clopidogrel and atorvastatin. Treatment for dissection aims to prevent arterial rupture and further ischaemia and infarction while preserving blood supply to the associated vascular territory [20]. Current guidelines recommend initial therapy with antiplatelets and/or anticoagulants [21]. The randomized CADISS clinical trial showed no significant difference between antiplatelet and anticoagulant treatments in rates of further ischaemic stroke, subarachnoid haemorrhage, and death at 3 months [22]. To date, symptomatic intracranial artery dissection, including vertebral artery dissection, is a significant challenge for clinicians due to the evidence of poor clinical outcomes and limited conservative and interventional management [23]. Since the introduction of endovascular treatments for intracranial artery dissection two decades ago, it has evolved from simple coiling or balloon occlusion for parent arterial sacrifice to more selective and delicate reconstruction styles, including overlapping stents, stent-assisted coils, and flow diverters [23]. As reported in a previous meta-analysis, deconstructive treatments were commonly utilized for vertebrobasilar dissections with good angiographic outcomes [24].

In conclusion, we reported a case of AHL and vertigo due to isolated cerebellar hypoperfusion without infarction. If a brain MRI, including PWI, was not performed in this case, the diagnosis and treatment of cerebellar ischaemia may have been delayed. Therefore, it is necessary to consider the possibility of a central cause in patients with AHL and vertigo, and it is important to confirm it through brain MRI, including PWI, and magnetic resonance angiography (MRA).

## Data Availability

Not applicable.

## Abbreviations

SSNHL: Sudden sensorineural hearing loss
VA: Vertebral artery
AHL: Acute hearing loss
HIT: Head impulse test
PTA: Pure-tone audiogram
DWI: Diffusion-weighted imaging
PWI: Perfusion-weighted imaging
PICA: Posterior inferior cerebellar artery
AICA: Anterior inferior cerebellar artery
hsCRP: High-sensitivity C-reactive protein
dB: Decibel
MRI: Magnetic resonance imaging
MRA: Magnetic resonance angiography
CTA: Computed tomography angiography
CT: Computed tomography
VBIS: Vertebrobasilar ischaemic stroke
VBI: Vertebrobasilar insufficiency
ATVS: Acute transient vestibular syndrome
